# The right ventricular involvement in dilated cardiomyopathy: prevalence and prognostic implications of the often-neglected child

**DOI:** 10.1007/s10741-022-10229-7

**Published:** 2022-03-22

**Authors:** Paolo Manca, Vincenzo Nuzzi, Antonio Cannatà, Matteo Castrichini, Daniel I. Bromage, Antonio De Luca, Davide Stolfo, Uwe Schulz, Marco Merlo, Gianfranco Sinagra

**Affiliations:** 1 Division of Cardiology, Cardiovascular Department, Azienda Sanitaria Universitaria Integrata Giuliana Isontina (ASUGI), University of Trieste, Via Valdoni 7, 34149 Trieste, Italy; 2 Department of Cardiovascular Science, Faculty of Life Science and Medicine, King’s College London, London, UK; 3 Division of Cardiology, Department of Medicine, Karolinska Institutet, Stockholm, Sweden; 4 Department of Cardiac Surgery, Heart Center, University of Leipzig, Leipzig, Germany

**Keywords:** Dilated cardiomyopathy, Right ventricle, Prognosis, Cardiac imaging, Advanced heart failure

## Abstract

Dilated cardiomyopathy (DCM) is a primary heart muscle disease characterized by left or biventricular systolic impairment. Historically, most of the clinical attention has been devoted to the evaluation of left ventricular function and morphology, while right ventricle (RV) has been for many years the forgotten chamber. Recently, progresses in cardiac imaging gave clinicians precious tools for the evaluation of RV, raising the awareness of the importance of biventricular assessment in DCM. Indeed, RV involvement is far from being uncommon in DCM, and the presence of right ventricular dysfunction (RVD) is one of the major negative prognostic determinants in DCM patients. However, some aspects such as the possible role of specific genetic mutations in determining the biventricular phenotype in DCM, or the lack of specific treatments able to primarily counteract RVD, still need research. In this review, we summarized the current knowledge on RV involvement in DCM, giving an overview on the epidemiology and pathogenetic mechanisms implicated in determining RVD. Furthermore, we discussed the imaging techniques to evaluate RV function and the role of RV failure in advanced heart failure.

## Introduction

Dilated cardiomyopathy (DCM) is a primary heart muscle disease defined as the impairment of left or biventricular systolic function, often associated with dilation, in absence of coronary artery disease or valvular heart disease [1]. Left ventricular (LV) morphology and function have been the predominant field of interests of clinicians, while a relatively few evidence was available about the significance of right ventricular (RV) involvement in DCM [2, 3]. Indeed, the right ventricle (RV) has been the “forgotten” chamber in the evaluation of DCM and, more in general, heart failure (HF) patients. This depends on various reasons and might be partially explained by the challenges imposed by RV anatomy and function, with clear limitations affecting its evaluation.

However, in the last years, we have faced a new renaissance for the RV. Progresses in cardiac imaging, mainly due to the advent of cardiac magnetic resonance (CMR) and the implementation of speckle tracking and 3D echocardiography, showed that RV involvement is common in DCM [2, 4–8], carrying important prognostic and therapeutic implications [2, 3, 9]. These elements highlight the importance of an accurate and serial evaluation of RV in all DCM patients, with a particular focus on its dynamic behavior during the course of the disease [3, 10].

In this review, we highlight the epidemiology, the pathogenetic mechanisms, and the possible best management of RV involvement in DCM patients. Furthermore, we discussed the imaging techniques to evaluate RV function and the role of RV failure in advanced heart failure. We also focused on the imaging techniques available for a multimodality RV assessment, along with the prognostic implications of RV impairment in specific settings of DCM.

### Epidemiology and pathogenesis

The prevalence of right ventricular dysfunction (RVD) in DCM varied in previous reports, ranging from 20 to 65%, and approaching 30% in the most recent series [2, 3, 8–10] (Table 1). Genetics play a key role in DCM but, apparently, the prevalence of RVD at diagnosis is similar between genetic and non-genetic forms [10]. Importantly, during the follow-up, a significant percentage of patients improve their RV function and the prevalence of RVD is generally lower in the natural history of the disease, with approximately 80% of patients normalizing their RV function under guideline medical treatment (GDMT) [3].

**Table 1:** Available evidences on the epidemiology and relative prognostic impact of right ventricular dysfunction in dilated cardiomyopathy

	**Study population**	**RVD definition**	**% of RVD**	**Outcome measure**
la Vecchia et al. [8]	92 DCM patients	RVEF < 35% (RV angiography)	58/92 (65%)	Not assessed
Gulati et al. [2]	250 DCM patients	RVEF < 45% (CMR)	86/250 (34%)	RVD associated with higher risk of D/HT (HR 3.90, 95% CI 2.16–7.04)
Venner et al. [9]	136 DCM patients	TAPSE ≤ 15 mm (Echo)	34/136 (25%)	RVD associated with higher risk of major CV events (HR 3.2, 95% CI 1.3–7.6)
Merlo et al. [3]	512 DCM patients	RVFAC < 35% (Echo)	103/512 (20%)	RVD associated with higher risk of D/HT (HR 1.71, 95% CI 1.02–2.85)
Pueschner et al. [5]	423 DCM patients	RVEF < 35% (CMR)	84/423 (19.8%)	RVD associated with higher risk of CV death (HR 3.00, 95% CI: 1.99–4.51)
Becker et al. [6]	216 DCM patients	RVEF < 45% (CMR)	83/216 (38%)	RVD associated shorter time to D/VA (HR 3.19, 95% CI 1.49–6.84)
Manca et al. [10]	104 genetically determined DCM patients	RVFAC < 35% (Echo)	30/104 (28.8%)	Not assessed

*DCM* dilated cardiomyopathy, *RVEF* right ventricular ejection fraction, *RV* right ventricular, *RVD* right ventricular dysfunction, *CMR* cardiac magnetic resonance, *TAPSE* tricuspidal annulus plane systolic excursion, *D/HT* all-cause death/heart transplant, *CV* cardiovascular, *VA* ventricular arrhythmias

**Fig. 1:**
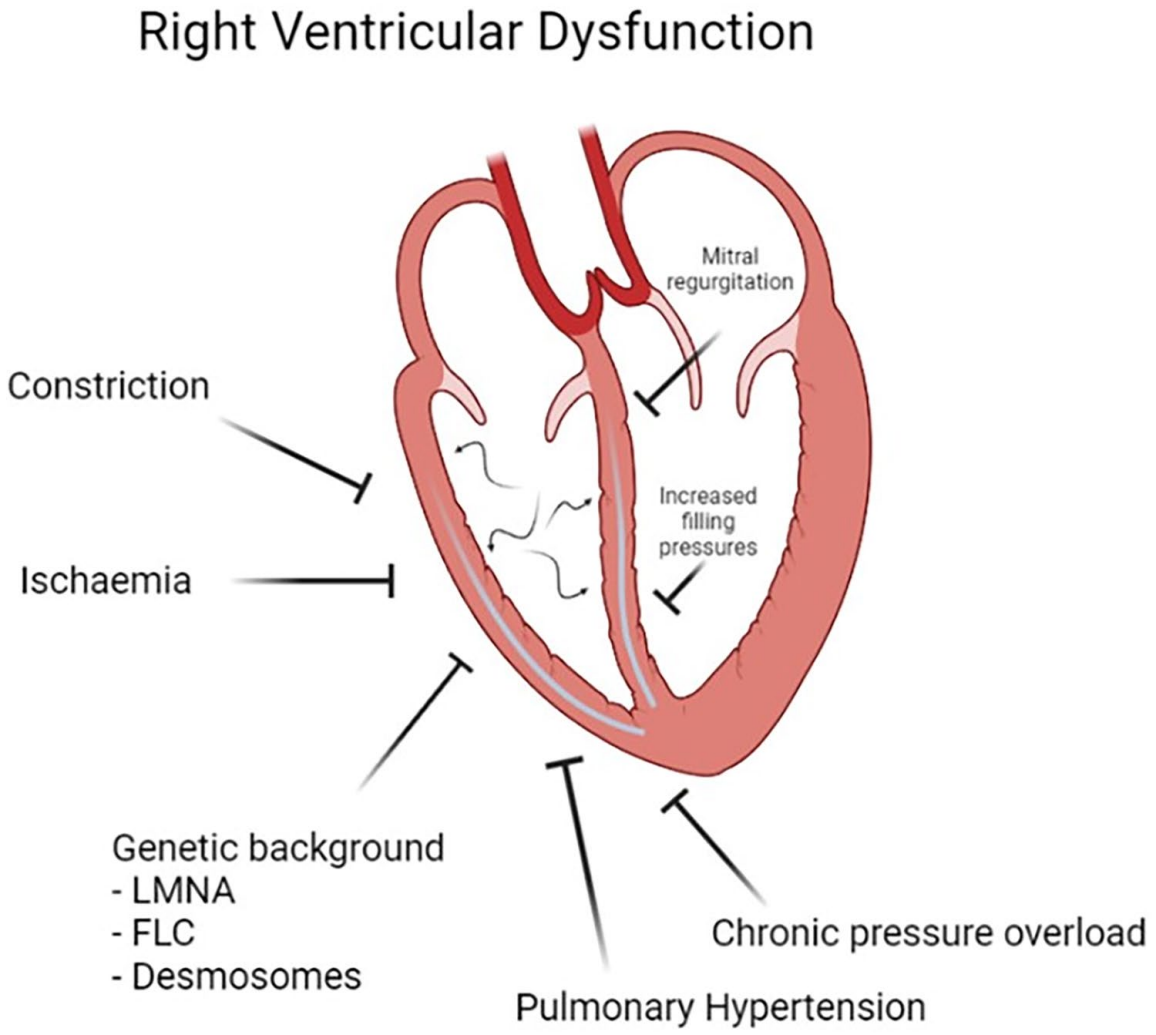
Mechanisms involved in determining right ventricular dysfunction in patients affected by non-ischemic dilated cardiomyopathy

Several mechanisms might be involved in the pathogenesis of RVD in DCM (Fig. 1). The most common one is related to the complex interventricular relationship. The systo-diastolic impairment of the LV, increased LV filling pressure, and relevant mitral regurgitation (MR) might in turn increase the pulmonary pressure. These factors lead to a type 2 pulmonary hypertension and consequently chronic pressure overload of the RV, which culminates in RV dilation and dysfunction [11]. Optimizing loading conditions with the implementation of loop diuretics and counteracting the detrimental activation of the sympathetic and renin-angiotensin system with GDMT usually have a positive effect on the RV function [12].

While the latter mechanism is common in all forms of heart failure with reduced ejection fraction (HFrEF), in DCM the presence of RVD might also be determined by a primary cardiomyopathic process, leading to an impaired RV myocardial contractility [13]. This specific mechanism could probably be more frequent in some specific genetic etiologies of DCM, such as pathogenic mutations in desmosomal genes, Desmin (*DESM*), Filamin C (*FLNC*), and Lamin (*LMNA*) genes, leading to an overlap between the DCM phenotype and specific forms of arrhythmogenic cardiomyopathy (AC) with biventricular involvement [14, 15]. RVD is usually involved in the so-called AC, irrespectively from the hemodynamic status and LV impairment, or it can develop during the long term of the disease as a progress of the cardiomyopathic process [15].

Further contributors to RVD may be the decreased RV coronary perfusion by a failing LV [16], as well as LV dilation in a restricted pericardial compartment that may limit RV diastolic function [17].

All these aforementioned mechanisms are not mutually exclusive. A complex interplay can be present in the same patient and a comprehensive evaluation is therefore mandatory to implement individualized solutions.

#### Role of cardiac imaging in the assessment of right ventricular function

Great progresses in cardiac imaging have been observed in the last years relatively to the RV function assessment (Table 2). In the past, the evaluation of RV was limited to gross data derived from chest X-ray [18] or to those derived by invasive, time-consuming, and radiation-based procedures such as RV angiography or radionuclide-based techniques [19].

**Table 2:** Advantages and disadvantages of mostly adopted techniques for right ventricular assessment

**Method**	**Advantages**	**Disadvantages**
Standard echocardiography	• Wide availability • Well-standardized measures • Acceptable intra-operator and inter-operator reproducibility • Hemodynamic assessment • Low cost • Follow-up assessment	• Several parameters (i.e., TAPSE, S’, RVFAC) not constantly in agreement among them • Poor correlation with CMR 3D RVEF • Unfeasible correlation with 3D RV assessment due to the particular RV shape and position
Advanced echocardiography	• 3D assessment of RV • Increased sensibility compared to standard evaluation • Higher correlation with CMR 3D RVEF	• Needing for skilled operators • Higher cost • Dedicated software • Different vendors • Underestimation of RV volumes
Cardiac magnetic resonance imaging	• Aetiological definition • High accuracy in RV volumes and function estimation • Very high sensibility in the detection of RVWMA • Tissue assessment (fat infiltration/replacement) • High intra-operator and inter-operator reproducibility	• High cost, time-consuming • Low availability in non-tertiary centers • Contraindicated in claustrophobic patients • Poor imaging quality in patients with device/arrhythmias
Ventriculography and thermodilution	• Functional measurements • Evaluation of acute response to drug administration • High reproducibility • Regional wall abnormality detection	• Invasive technique • Iodium contrast needing • Radiation exposure • Needing for trained professionals
Radionuclide	• 3D RVEF evaluation • Good inter-operator reproducibility • Prognosticator in HFrEF	• Motion artifacts • Low-quality images in presence of arrhythmias • Feasible only in experienced centers

*TAPSE* tricuspidal annulus excursion, *S’* tricuspid lateral annular systolic velocity, *RVFAC* right ventricular fraction area change, *CMR* cardiac magnetic resonance, *RVEF* right ventricular ejection fraction, *RV* right ventricle, *RVWMA* right ventricular wall motion abnormalities

Standard echocardiography provides clinicians with a simple, fast, non-invasive, and widely available method to study RV function. However, the anterior position in the chest of RV, its complex geometric shape, and function limit the accuracy of the echocardiographic-derived measures [20].

The more frequently considered parameters in the evaluation of RV function are the tricuspidal annulus plane systolic excursion (TAPSE), the right ventricular fractional area change (RVFAC), and the tricuspid lateral annular systolic velocity (S’).

TAPSE evaluates the longitudinal RV function measured with the M-mode in the apical 4-chamber view [21]. A value of less than 1.7 cm indicates RV dysfunction [22]. Although widely adopted and simple to measure, it shows clear limitations, reflecting only the longitudinal function of the basal RV wall, possibly overestimating or underestimating RVD in specific settings [23]. Specifically, the value of TAPSE in patients who underwent previous cardiac surgery is usually underestimated and it manifests important drawbacks in this setting [24].

RVFAC is a 2D measure obtained in the RV-focused apical 4-chamber view by manually tracing the endocardial RV borders at end-diastole and end-systole [22]. In comparison to TAPSE, RVFAC offers a more comprehensive evaluation of RV function, even though the RV outflow tract is not considered due to the particular anatomy of RV. A value ≥ 35% of RVFAC is considered normal in adults [22]. An important limitation to the assessment of RVFAC is represented by the prominent trabeculations of the RV, making border definitions particularly challenging [20]. However, previous studies showed that RVFAC might be the 2D echocardiographic parameter with the best correlation with RVEF determined by CMR [25, 26].

The S’ velocity reproduces the tissue Doppler systolic velocity of the tricuspid annulus, being similar to TAPSE, a measure of longitudinal RV function [23]. A value of S’ < 9.5 cm/s is considered the cut-off to identify RVD. It presents the same limitations of TAPSE, including angle and load dependency [27].

Among the abovementioned parameters, S’ appears to be the best potential earlier marker of RVD [28].

Another advantage of standard echocardiography is the possible simultaneous acquisition of hemodynamic information of the right heart through Doppler flow measurements [23]. Of interest is the systolic pulmonary artery pressure (sPAP) that might be estimated by the tricuspid regurgitation (TR) peak velocity [23]. This information could be crucial in differentiating RVD derived from high LV filling pressure (in case of high sPAP, typically ischemic heart disease) or RVD derived from direct cardiomyopathic RV involvement (possible presence of low sPAP) [8].

### Advanced echocardiography and cardiac magnetic resonance

Recently, new techniques of advanced echocardiography have shown promising potential value in implementing the information derived from standard echocardiography. Speckle tracking two-dimensional (2D) analysis is able to detect subtle abnormalities of RV function in presence of normal standard echocardiographic assessment, and it is relatively load and angle independent compared to standard parameters [29–31]. Previous experiences suggested that right ventricular global longitudinal strain (RVGLS) has better correlation with RVEF calculated by CMR and with major cardiovascular (CV) outcomes compared to standard echocardiographic measurements [32, 33]. Furthermore, in non-selected HFrEF cohorts with apparently normal RV function, subtle functional abnormalities detected by depressed RVGLS carry negative consequences in terms of CV outcomes [34].

**Fig. 2:**
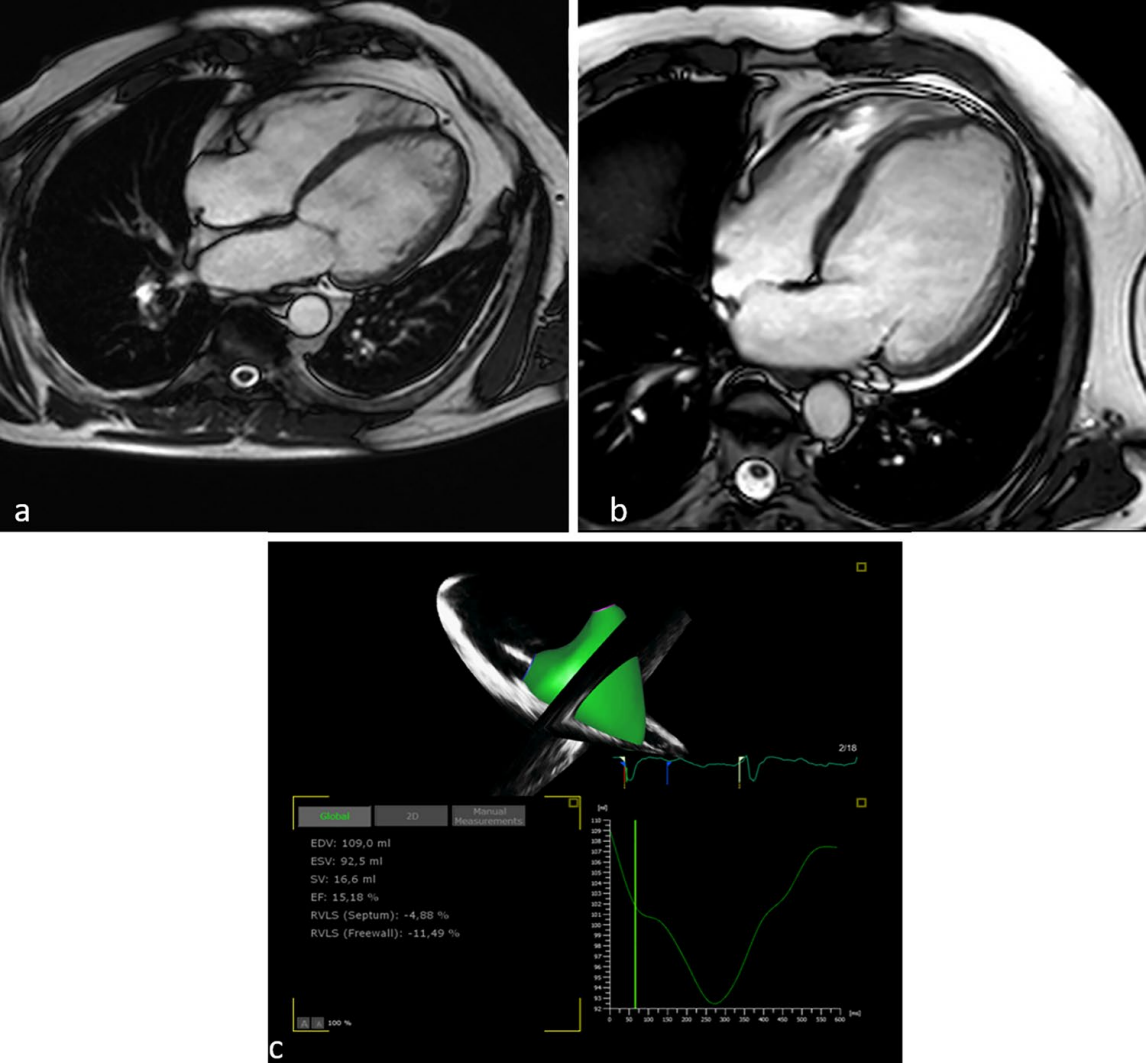
Cardiac magnetic resonance and 3D echocardiography evaluation of right ventricle. **a** A dilated right ventricle in a patient affected by dilated cardiomyopathy and biventricular dysfunction. **b** A dilated cardiomyopathy without involvement of right ventricle. **c** A severely depressed right ventricular function analyzed with 3D echocardiography

Three-dimensional (3D) echocardiography overcomes 2D geometric assumptions, integrating the longitudinal, radial, and antero-posterior components of RV contraction [35]. When validated against CMR, it might represent an important alternative for the assessment of RVEF [36] (Fig. 2). Nevertheless, RV volumes are usually underestimated using this technique compared to CMR [36, 37]. Indeed, especially in the presence of dilated RV and broad distance from the probe to RV wall, a wider band of hues inside the RV wall is seen, and manual tracking might exclude this blurred area resulting in underestimation of the real RV cavity [38].

CMR represents actually the gold standard for the assessment of RV function and morphology [28]. Indeed, the multiplanar imaging and 3-dimensional volume acquisition obtained by CMR overcomes the need for geometric assumptions regarding RV shape that limits the accuracy of standard echocardiography [39, 40]. Furthermore, balanced steady-state free-precession cine acquisitions ensure high-spatial and time resolution images, with accurate discrimination between blood and endocardium [39]. Compared to other techniques, CMR shows high accuracy and reproducibility in the estimate of RV volumes and RVEF with good interobserver and intraobserver variability [41–43]. Robust evidences exist on the prognostic value of RVEF determined by CMR in DCM [2, 5]. However, while most of the studies found that an RVEF value < 45% is associated with negative CV outcomes, less agreement is available on the RVEF cut-off to define RVD [44, 45]. The other major advantage of CMR is the capability of morphological assessment and tissue characterization beyond RV function. T2-weighted short-tau inversion recovery (T2w-STIR) imaging using an electrocardiogram-gated triple inversion recovery (IR) theoretically is able to depict tissue edema, and might represent a useful diagnostic tool when an overlapping active inflammatory process is suspected, such as in acute or chronic myocarditis or cardiac sarcoidosis [46]. The presence of late gadolinium enhancement at the right RV insertion site has been reported in a variable number of DCM patients studied with CMR, but its significance in terms of prognosis remains uncertain and probably neutral [47, 48]. However, the real prevalence of late gadolinium enhancement in the RV is complicated by the thin wall, which might limit the accuracy in the assessment of possible areas of scar [49]. Nevertheless, due to some major limitations of CMR such as its limited availability, the request of patient compliance, and the troubles of performing the exam in patients wearing a cardiac device, most centers still use echocardiography for the routine assessment of RV.

### The importance of a longitudinal evaluation

The concept of longitudinal evaluation in DCM has gained progressively more importance in the last years [50]. Progresses in medical therapy not only dramatically reduced mortality in DCM [51], but also showed the capability of positively counteracting the adverse cardiac remodeling, potentially restoring a normal cardiac shape and function in a substantial number of patients [52]. This phenomenon has been extensively studied in the LV, demonstrating that approximately one-third of patients affected by DCM might undergo through a left ventricular reverse remodeling (LVRR) up to 24 months since the diagnosis, with positive consequences in terms of cardiac outcomes [53, 54].

This capability of cardiac remodeling has been less extensively studied in the RV. In the largest DCM population with available periodical reassessment of RV function with standard echocardiography, approximately 80% of patients with baseline RVD demonstrated a normalization of RV function in the following 6–12 months, with an important predictive value of a subsequent LVRR [3]. Importantly, in another study focused on DCM patients with available genetic data, the percentage of patients normalizing RV function during follow-up was high (≃70%), with similar findings among patients with genetic negative or *TTN*-determined DCM, while patients affected by other genetic etiologies had a lower probability to improve their RV function, highlighting a possible different pathogenetic mechanism of RVD in these patients [10]. Importantly, RVD might also develop in advanced stages of the disease in patients with baseline normal RV function, conferring negative prognostic value [3, 10].

Currently, echocardiography remains the method of choice for periodical revaluation of cardiac function in DCM, and a particular focus on RV function should be always encouraged during the follow-up of these patients.

#### Prognostic implications of right ventricular dysfunction and specific therapeutic challenges

Prognostic stratification remains a cornerstone in the management of DCM patients. Among the other parameters, RV involvement, both in terms of morphological and functional alterations, has been proven to be one of the strongest negative outcome modifiers, particularly when combined with concomitant pulmonary hypertension [55].

A reduced benefit from GMDT in terms of LV improvement has been described in DCM patients with persistent RVD [3], reflecting perhaps a more advanced disease. At the same time, the presence of RVD negatively affected the response to CRT [56, 57] and mitral valve repair [58–60] in HFrEF of mixed origin, including DCM patients.

Potentially, all reports considering RVD have been variably associated with adverse outcomes. In a previous experience including approximately one hundred DCM patients, a TAPSE ≤ 14 mm was significantly associated with a higher risk of all-cause death (ACD) or heart transplantation (HTx) [61]. Interestingly, even in patients with normal TAPSE, a depressed RV free wall strain is associated with higher risk of CV outcomes [33].

Analogously, a depressed value of RVFAC, both at the initial clinical presentation and at follow-up evaluation, was also associated with higher risk of HTx and ACD in a large series of patients affected by DCM [3].

A reduced RVEF determined by 3D echocardiography was associated with poor CV outcome in patients affected by various CV diseases [62]. Furthermore, recent data showed that an RVEF < 43% determined by 3D echocardiography could predict a worse cardiovascular outcome in DCM [7].

The most robust evidence of the prognostic role of RVD in DCM comes from studies which evaluated RVEF with CMR. In a study of 250 prospectively enrolled DCM patients, Gulati et al. demonstrated a strong and independent role of RVD in determining ACD, HTx, or heart failure hospitalizations (HFH) [2]. Similarly, the presence of RVEF < 45% was associated with a sevenfold increased risk of ventricular arrhythmias and a shorter time to all-cause mortality in more than two hundred DCM patients [6]. In another population of more than four hundred patients affected by DCM, a depressed RVEF was associated with a fivefold increased risk of ACD [5]. Finally, RVGLS analyzed by CMR was demonstrated to show an incremental prognostic role in reclassifying the risk of major cardiovascular events in recent series of DCM patients [63].

All these data taken together show the importance of a comprehensive RV assessment in the prognostic stratification of DCM patients.

Further research is needed to provide a direct comparison of the different RV imaging parameters in the prognostic stratification of patients with DCM and RVD.

#### Specific considerations in DCM with advanced heart failure

In patients with DCM and advanced HF, specific therapies, namely mechanical long-term support or HTx, must be considered [64]. In this setting, RV assessment is crucial, as it implies different approaches and profoundly changes the management of these patients.

In case of indications to left ventricular assist device (LVAD), acute RV failure post LVAD implantation occurs in 13–40% of patients, conferring important prognostic implications [65, 66].

Acute RV failure post LVAD implantation is defined by INTERMACS as need of an RVAD or requirement of inhaled nitric oxide or inotropic therapy for more than 1 week any time after LVAD implantation in the presence of symptoms and signs of persistent RVD, such as central venous pressure > 18 mmHg with a cardiac index < 2 L/min/m2 in the absence of elevated left atrial or pulmonary capillary wedge pressure (PCWP) (> 18 mmHg), cardiac tamponade, ventricular arrhythmias, or pneumothorax [67].

**Fig. 3:**
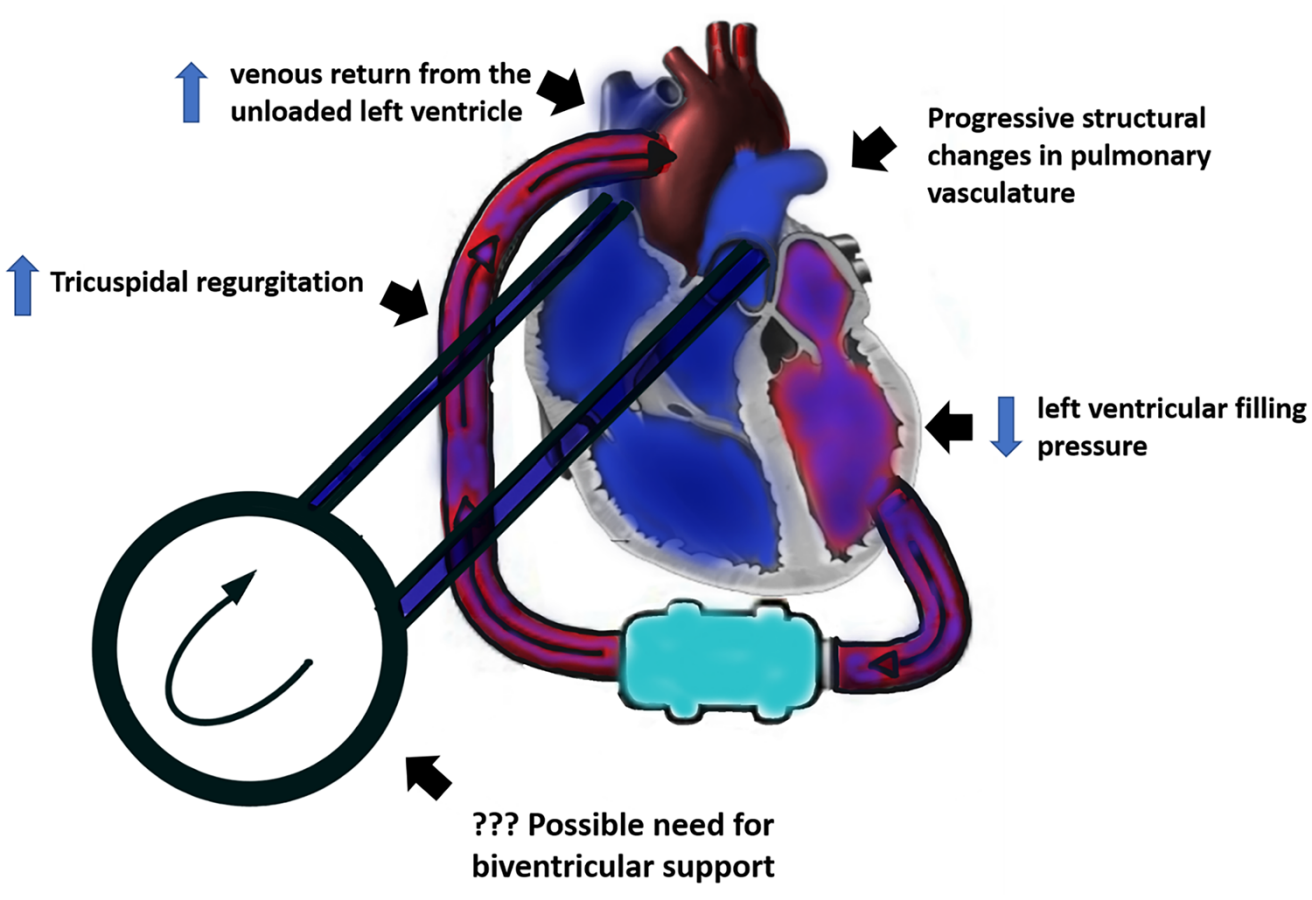
The complex pathogenetic interplay of right ventricular failure post left ventricular assist device implantation

The mechanism of this phenomenon is not completely understood, but it is mostly related to the acute hemodynamic change post LVAD implantation (Fig. 3). Indeed, the increased RV preload due to the augmented venous return from the unloaded LV can further increase the physiologic burden on an RV that may already be dysfunctional pre-LVAD and can potentially exacerbate tricuspid regurgitation [68]. This mechanism is only partially balanced by the decreased RV afterload due to the reduction of LV filling pressures, while the possible structural changes in pulmonary vasculature require longer to develop [69]. At the same time, possible ineffective unloading of the LV under LVAD can also be detrimental for the RV and leads to RV failure [70]. An optimal hemodynamic balance is thus complicated in these patients, and fluid status must always be optimized and continuously monitored [12].

On the other hand, RV failure might develop even with a late onset (i.e., > 5 weeks since LVAD implantation) in patients supported by LVAD [71]. This condition has been reported in a various number of LVAD patients, ranging from 8 to 45% in previous observations [71–74], and leading to a poor prognosis [75]. The pathogenesis of late-onset RVF is multifactorial, and it is due to a complex interplay between intrinsic RV function and hemodynamic changes determined by LVAD support [72]. Indeed, while it is plausible that late RVF could occur in a greater percentage of patients with pre-existing underestimated RVD, suction from LVAD and leftward shift of the interventricular septum could induce changes in RV morphology resulting in RV impairment even in patients without pre-existing RVD [76, 77]. Furthermore, other important complications of LVAD such as aortic valve regurgitation and ventricular arrhythmias have been advocated in the progression of late RVD, with possible benefits of aortic valve replacement in patients with symptomatic aortic valve regurgitation [78, 79].

Based on the above considerations, it appears clear that one of the main determinants of RV failure post LVAD implantation is the pre-implant RV function. An adequate pre-implant RV evaluation, comprehensive of echocardiographic parameters such as RV free wall strain, RVFAC, and RV/LV size ratio, as well as some parameters evaluating left atrium and LV should be always performed to predict the possibility of RVF and to consider the possibility of biventricular instead of LV only support [68, 80, 81]. However, even though many specific multiparametric scores have been proposed in this setting, many other studies have found no predictors of RV failure, especially in cases of late onset [77]. Currently, the role of dobutamine stress echocardiography in selecting patients for LVAD is not well established, but it has been proven that a positive response to dobutamine myocardial stress in patients with LVAD and decreasing support might be useful to identify patients who could be weaned from LVAD [82].

Although the presence of RVD represents a marker of advanced disease prompting an HTx instead of LVAD, RV failure might be seen also in patient post HTx, representing one of the major outcome determinants [83]. In these patients, the presence of high PVR and pulmonary artery pressure pre-transplant lead to pressure overload of the donor RV and, therefore, to RV failure [84]. Other factors that may contribute to RVD post-surgery might be inadequate or prolonged hypothermic preservation, ischemic RV injury on cardiopulmonary bypass, and the change in contractile pattern of RV post cardiotomy [85].

In both post LVAD and post HTx, patients with RVF might need an inotropic treatment to support the hemodynamic status due to the failing RV, with dobutamine, milrinone, and levosimendan as the most used agents, possibly associated with vasodilators such phosphodiesterase type-5-inhibitors to ensure a reduction of PVR and RV afterload [86–88]. In some refractory cases, the implementation of RV-specific mechanical supports should be considered [89].

#### Future perspectives

Although our knowledge on RV pathophysiology and our capability of its assessment have grown up in the last decades, many fascinating targets could be addressed in the next future.

It is plausible that CMR will be progressively more available, and its constant implementation, along with speckle tracking techniques in the routine assessment of RV, will probably contribute to a give real estimation of the RVD in DCM, helping clinicians in the prognostic stratification and therapeutical management.

At the moment, no specific therapies are present for RVD and the most important therapeutical step is to ensure an optimal hemodynamic status concurrently with the constant implementation of drugs for LV systolic dysfunction.

Progresses in the genetics field led us to study some specific therapies for some genetic etiologies such as *LMNA* and *PLN* [90–92]. Hopefully these therapies might change the natural history of these entities, and can directly counteract the cardiomyopathic process in both ventricles, representing an important alternative where the usual GDMT fails to improve symptoms and functional performance.

Finally, the advent of percutaneous correction of tricuspid regurgitation might represent a possible therapeutic target for some DCM patients where RVD could be the result of chronic volume overload which cannot be properly addressed by medical treatment [93].

## Conclusions

The importance of the RV function in patients affected by DCM is pivotal. Progresses in cardiac imaging showed that the prevalence is high in DCM in distinct phases of the disease, with many possible pathogenetic mechanisms. At the moment, specific treatments for RVD are still lacking and further research is advocated to implement pharmacotherapies with a precise target on RV.

## Data Availability

Not applicable.
